# Patterns and characteristics of maxillofacial fractures in women

**DOI:** 10.1007/s10006-022-01085-8

**Published:** 2022-06-09

**Authors:** Jason Diab, Mark H. Moore

**Affiliations:** 1 Australian Craniofacial Unit, North Adelaide, 72 King William St, Adelaide, SA 5006 Australia; 2 Royal Adelaide Hospital, Adelaide, Australia; 3 Women and Children’s Hospital, Adelaide, Australia; 4 School of Medicine, University of Notre Dame, Sydney, Australia; 5 University of Adelaide, Adelaide, Australia

**Keywords:** Female, Indigenous, Facial fractures, Accidental falls, Australia

## Abstract

**Purpose:**

Facial trauma in women is complex with physical, psychosocial, and cultural influences impacting clinical presentations. Although multifactorial, assaults and falls are principally reported as the main causes.

**Methods:**

A retrospective review was conducted from January 2012 to January 2017 at the Women and Children’s Hospital and Royal Adelaide Hospital, Adelaide. All maxillofacial fractures in women that attended or were referred to the unit were included in this study. The primary objective was to analyse epidemiological trends of facial fractures and clinical outcomes in the South Australian female population.

**Results:**

There is a bimodal distribution of facial fractures at 25–35 years and 65 + years. Indigenous females were 19.5 years younger than non-indigenous females (30.5 vs 49.9, *P* < 0.001). Approximately half the cohort had a fall-related facial fracture, followed by assault (26.2%), and sports (10.3%). There was a higher proportion of non-alcohol-related trauma from assaults than alcohol-related assaults (72.5% vs 27.5%, *P* < 0.001). Over half (58.0%) of the cohort had a midface fracture. The elderly had increased odds of 1.9 fold for facial fractures in winter, largely from falls, compared to younger women. Associated injuries were present in almost half the elderly women with 2.6 times the risk compared to younger women. Younger women had higher incidences of surgical intervention (52.6% vs 14.3%, *P* < 0.05).

**Conclusions:**

Young women disproportionately experience larger incidences of non-alcohol-related assaults requiring operative intervention of the mandible, whereas elderly women principally suffer fall-related facial fractures with higher rates of associated injuries.

## Introduction

Facial fractures are largely experienced by young adult males. There is however unique profiling for women who experience facial trauma from childhood to elderly. Often overlooked in many studies, the complexity of facial fractures in women involves a more global assessment of physical and psychosocial wellbeing with potential secondary consequences to functional impairment and disfigurement. Domestic violence is a growing concern amongst developing and developed countries where the perpetrator is often intimately known to the patient adding clout to the mechanism of injury and potential risk factors. International studies have indicated that assaults are disproportionately experienced by indigenous persons from Canada, Australia, and New Zealand compared to their non-indigenous counterparts [1–3]. The impact of domestic violence against women and children in Australia has been estimated at $22 billion AUD [4]. In an ageing population, falls are increasingly common presentation and reason for hospitalisation. Elderly females are more prone to falls than men with increased risks of associated injuries and polytrauma.

There is scant data on female maxillofacial fractures internationally, yet alone in Australia, that focuses specifically on the risk profile and clinical outcomes. The Women and Children’s Hospital and Royal Adelaide Hospital is the main tertiary and quaternary referral service for paediatric and adult facial fractures in South Australia, Australia, with care extending to parts of Northern Territory, rural New South Wales and Victoria. Care is delivered through inpatient, ambulatory care and outreach services treating approximately 600 patients yearly [5]. The main objective was to analyse the epidemiology of facial fractures in South Australian females with particular focus on vulnerable groups and differences in clinical outcomes. This study represents the first statewide comprehensive analysis of the South Australian experience focusing on the patterns and trends of facial trauma and clinical outcomes in women.

## Methods

A retrospective institutional review was conducted at the Women and Children’s Hospital and Royal Adelaide Hospital, Adelaide, Australia, from January 2012 to January 2017 for all patients who sustained facial fractures. Patients were assessed by a plastic surgical trainee and/or craniofacial surgeon at the time of presentation. According to the national census in 2016, approximately 1.7 million people lived in South Australia with 50.7% females [6]. The data was retrospectively reviewed from medical records, progress notes, imaging, and operative notes. Ethics approval was granted from the Human Research and Ethics Committee [HREC/17/RAH/402]. The primary objective was to analyse epidemiological trends of facial fractures in the female population. The secondary objective was to determine differences in trends and clinical outcomes amongst younger females (18–65 years) and elderly (65 years and above).

The following recorded parameters were analysed for this project as per our standardised registry:

*Social demographics*: age, age groups, indigenous status, and alcohol intoxication.^.^ *Age groups* included < 18 years, 18–25, 26–35, 36–45, 46–55, 56–65, and 65 + (elderly).

*Socioeconomic parameters and timing of injury*: season, timing of injury, postcode, socioeconomic index for areas (SEIFA), and Australian statistical geography standard (ASGS) scale. The *Socioeconomic Indexes for Areas (SEIFA)* is a measure of disadvantage created by the Australian Bureau of Statistics (ABS) who defines relative socioeconomic advantage and disadvantage in terms of people’s access to material and social resources and the ability to participate in society. The SEIFA score was assessed from the patient’s postcode and analysed in conjunction with the ABS framework. The score of the residential statistical local area of each person was used as the area-based composite measure of socioeconomic status from the index of relative socioeconomic advantage and disadvantage (IRSAD) [7]. Overseas adults were excluded from the socioeconomic parametric analysis. The *Australian Statistical Geography Standard (ASGS)* defines Remoteness Areas into five classes of relative remoteness across Australia: major cities, inner regional, outer regional, remote, and very remote. This index uses distances to population centres as the basis for quantifying service access [8]

*Aetiology and type of injury*: mechanism of injury (assault, road traffic accident (RTA), sports, falls, work, other), type of injury [9] (orbit, orbitozygomatic, mandible, zygomatic arch, nasal, naso-orbito-ethmoidal (NOE), frontal sinus, panfacial, dentoalveolar middle cranial base, posterior cranial base), multiple fractures, recurrent fractures, and associated injuries.

*Treatment, complications, and hospitalisation*: treatment [conservative, surgery, open reduction internal fixation (ORIF)], complications, re-operations, and length of stay (LoS).

A statistical analysis using SPSS (Version 26, IBM Corporation, Armonk, NY, USA) was computed for continuous variables assessing the relationship between linear data and correlation based on a level of significance set at *P* value of 0.05. Continuous variables were expressed as mean, median and standard deviation (SD). A paired independent *t* test and Pearson chi test were conducted to assess differences between young females (18–65 years) and elderly females for continuous and categorical variables. A binary and multivariate logistic regression analysis was conducted to analyse odds between age groups expressed as odds ratios (ORs) and 95% confidence intervals (95% CI).

## Results

### Demographic

Of 2559 patients from 2012 to 2017, there were 583 females who presented with facial fractures (22.8%) (Table 1). The average age was 48.6 years with a bimodal distribution (Shapiro–Wilk test < 0.001) peaking at 25–35 years (13.6%) and 65 + years (32.4%). The indigenous female population rate of facial fractures was 6.7% ranging from 3–54 years (Shapiro Wilk test = 0.799). Indigenous females were 19.5 years younger than non-indigenous females (30.5 vs 49.9, *P* < 0.0001). The most prevalent season for facial fractures was autumn (26.6%). Alcohol consumption at the time of injury was reported in 66 patients (11.3%). The most frequented time for presentation to an emergency department occurred in the morning and afternoon (29.5%), respectively. The most disadvantage socioeconomic group had the highest incidence of facial fractures (34.0%). Approximately two thirds of persons from the study population largely represented facial fractures from major cities.

**Table 1:** Summary of female patient profile and clinical outcomes

		*N* (%)
	Total	583
Demography		
Age (years)	Mean (SD) Range	48.6 (26.2) 0–98
Age groups	< 18 years	69 (11.8%)
	18–25 years	72 (12.3%)
	25–35 years	79 (13.6%)
	35–45 years	74 (12.7%)
	45–55 years	60 (10.3%)
	55–65 years	40 (6.9%)
	65 years +	189 (32.4%)
Alcohol	Yes	66 (11.3%)
Indigenous	Yes	39 (6.7%)
Timing of injury		
Seasons	Summer	130 (22.3%)
	Autumn	155 (26.6%)
	Winter	149 (25.6%)
	Spring	149 (25.6%)
Timing	Morning (0600–1200)	172 (29.5%)
	Afternoon (1201–1659)	172 (29.5%)
	Evening (1700–1959)	94 (16.1%)
	Night (2000–0559)	145 (24.9%)
Year	2012	106 (18.2%)
	2013	88 (15.1%)
	2014	111 (19.0%)
	2015	78 (13.4%)
	2016	93 (16.0%)
	2017	107 (18.4%)
Socioeconomic parameters		
IRSAD quintiles	Quintile 1 (most disadvantage)	196 (34.0%)
	Quintile 2 (more disadvantage)	72 (12.5%)
	Quintile 3 (middle disadvantage)	129 (22.4%)
	Quintile 4 (less disadvantage)	116 (20.1%)
	Quintile 5 (least disadvantage)	64 (11.1%)
Australian Statistical Geography Standard (ASGS)	Major city	387 (66.4%)
	Inner regional	71 (12.2%)
	Outer regional	86 (14.8%)
	Remote	19 (3.3%)
	Very remote	4 (0.7%)
Aetiology		
Type of sport	Assault	153 (26.2%)
	Fall	281 (48.2%)
	Sports	60 (10.3%)
	Road traffic accident (RTA)	55 (9.4%)
	Work	5 (0.9%)
	Animal-related	27 (4.6%)
	Other	2 (0.3%)
Type of injury	Mandible	122 (20.9%)
	Orbitozygomatic	176 (30.2%)
	Orbit	162 (27.8%)
	Nasal	70 (12.0%)
	Zygomatic arch	15 (2.6%)
	Frontal Sinus	12 (2.1%)
	NOE	7 (1.2%)
	Dentoalveolar	11 (1.9%)
	Middle cranial base	3 (0.5%)
	Pan facial	5 (0.9%)
	Posterior cranial base	0 (0.0%)
Mandible	Condyle	46 (39.0%)
	Coronoid process	2 (1.7%)
	Ramus	2 (1.7%)
	Angle	22 (18.6%)
	Body	13 (11.0%)
	Symphyseal	33 (28.0%)
Multiple fractures	1	486 (83.4%)
	> 1	97 (16.6%)
Associated injuries	Yes	188 (32.2%)
Cervical spine fracture	Yes	14 (2.4%)
Recurrent fracture	Yes	18 (3.1%)
Treatment		
Surgery	Yes	224 (38.4%)
	No	359 (61.6%)
ORIF	Yes	116 (19.9%)
Complications	Yes	27 (4.6%)
Re-operations	Yes	11 (1.9%)
Length of stay (days)	Mean (SD)	3.80 (15.0)
	Median	1.00

Approximately half the cohort had a fall-related facial fracture, followed by assault (26.2%), and sports (10.3%). Orbitozygomatic fractures were the most common type of facial fracture (30.2%) with the midface representing over half the cases. Multiple fractures were identified in 97 patients (16.6%). One hundred and eighty-eight (32.2%) patients had an associated injury attributed mainly by falls resulting in neurological injury (*n* = 36), soft tissue injury (*n* = 36), cardiovascular events (*n* = 31), upper and lower injuries (*n* = 26, *n* = 26), and other injuries. There were fourteen patients with a cervical spine fracture (seven falls, four assaults, two RTAs, and one sport). There were 18 recurrent fractures. The operative rate was 61.6% and the remaining 38.4% were managed conservatively with serial reviews. There were twenty-seven (4.6%) post-operative complications related to infection, plate exposure, enophthalmos, nerve injury, and analgesic opioids. There were eleven re-operations (five orbits, three mandibles, and three panfacials). The mean hospital LoS was 3.8 days (SD ± 15.0).

### Younger females and elderly females

**Fig. 1:**
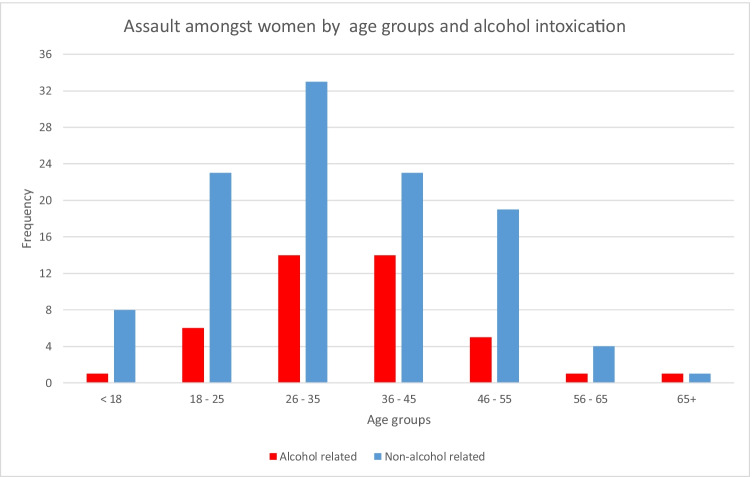
Assaults amongst women by age groups and alcohol intoxication

Younger females had a higher proportion of alcohol-related facial trauma compared to elderly females (18.8% vs 2.1%, Table 2). However, there was a higher proportion of non-alcohol-related trauma from assaults than alcohol-related assaults (72.5% vs 27.5%, *P* < 0.001, Fig. 1). There was a significant difference in proportions between types of fractures from assaults between young and elderly females with the midface commonly afflicted [orbit, *n* = 51; orbitozygomatic, *n* = 27] than mandible (*n* = 36, Table 2). The elderly had higher proportion of facial fractures in winter, largely from falls, with increased odds of 1.9 compared to younger women (Table 3). Young women from the most and more disadvantaged socioeconomic areas had greater proportions of facial fractures compared to elderly counterparts. Elderly women from the least disadvantage areas had 2.9 times the risk of a facial fracture compared to younger counterparts. Elderly women from major cities were 1.8 times more likely to have a facial fracture than younger counterparts. With increasing remoteness, younger women were more likely to have a facial fracture compared to their counterparts.

**Table 2:** A comparison of younger females and elderly females

	Younger females aged 18–65 years	Elderly females aged 65 years and above	*P* value
Demography			
Age (years) Mean ± SD	(*n* = 325) 37.9 ± 13.1	(*n* = 189) 80.1 ± 7.5	*P* < *0.001*
Indigenous Yes	34 (10.5%)	0 (0.0%)	*P* < 0.001
Alcohol Yes	61 (18.8%)	4 (2.1%)	*P* < *0.001*
Timing of injury			
Season Summer Autumn Winter Spring	74 (22.8%) 97 (29.8%) 66 (20.3%) 88 (27.1%)	40 (21.2%) 43 (22.8%) 62 (32.8%) 44 (23.3%)	*P* = 0.015
Socioeconomic parameters			
IRSAD quintiles Quintile 1 (most disadvantage) Quintile 2 Quintile 3 Quintile 4 Quintile 5 (least disadvantage)	125 (38.9%) 44 (13.7%) 74 (23.1%) 56 (17.4%) 22 (6.9%)	50 (26.7%) 13 (7.0%) 45 (24.1%) 46 (24.6%) 33 (17.6%)	*P* < 0.001
Australian Statistical Geography Standard (ASGS) Major city Inner regional Outer regional Remote Very remote	197 (60.6%) 38 (11.7%) 60 (18.5%) 10 (3.1%) 17 (3.3%)	139 (73.5%) 21 (11.1%) 22 (11.6%) 5 (2.6%) 1 (0.5%)	*P* = *0.013*
Aetiology			
Assault Fall Sports RTA Work-related Animal-related Other	142 (43.7%) 86 (26.5%) 35 (10.8%) 37 (11.4%) 3 (0.9%) 21 (6.5)% 1 (0.3%)	2 (1.1%) 175 (92.6%) 1 (0.5%) 8 (4.2%) 2 (1.1%) 1 (0.5%) 0 (0.0%)	*P* < *0.001*
Facial fractures Mandible orbitozygomatic Orbit Zygomatic arch Nasal NOE Frontal sinus Dentoalveolar Panfacial	80 (24.6%) 80 (24.6%) 93 (28.6%) 10 (3.1%) 40 (12.3%) 4 (1.2%) 8 (2.5%) 7 (2.2%) 3 (0.9%)	23 (12.2%) 85 (45.0%) 57 (30.2%) 3 (1.6%) 16 (8.5%) 1 (0.5%) 2 (1.1%) 2 (1.1%) 0 (0.0%)	*P* < 0.001
Mandible Condyle Coronoid process Ramus Angle Body Symphyseal	27 (33.3%) 2 (2.5%) 1 (1.2%) 19 (23.5%) 5 (6.2%) 27 (33.3%)	10 (43.5%) 0 (0.0%) 1 (4.3%) 1 (4.3%) 8 (34.8%) 3 (13.0%)	*P* < 0.001
Multiple fractures 1 fracture > 1 fracture	261 (80.3%) 64 (19.7%)	167 (88.4%) 22 (11.6%)	*P* = *0.018*
Associated injuries	81 (24.9%)	88 (46.6%)	*P* < *0.001*
Cervical spine fracture	7 (2.2%)	7 (3.7%)	*P* = *0.298*
Recurrent fracture	15 (4.6%)	3 (1.6%)	*P* = 0.033*
Treatment			
Surgery Yes No	171 (52.6%) 154 (47.4%)	27 (14.3%) 162 (85.7%)	*P* < *0.001*
ORIF	90 (27.7%)	15 (7.9%)	*P* < *0.001*
Complications	21 (6.5%)	5 (2.6%)	*P* = *0.057*
Re-operation	9 (2.8%)	1 (0.5%)	*P* = 0.076
Length of stay (days) Mean ± SD	4.0 ± 18.9	4.0 ± 7.3	*P* = *0.961*

^*^Italicised values indicate statistical significance, *P* < 0.05; #, no numerical value

**Table 3:** Univariate and multivariate logistic regression for young females and elderly females

	Univariate analysis: facial fracture odds for elderly to young females (95% CI)	Multivariate analysis: facial fracture odds for elderly to young females (95% CI)
Socioeconomic parameters and timing of injury		
Alcohol	0.094 (0.033–.0262)*	-
Season Summer Autumn Winter Spring	0.911 (0.590–1.406) 0.692 (0.457–1.048) *1.916 (1.276*–2.877)* 0.817 (0.539–1.240)	-
IRSAD quintiles Quintile 1 (most disadvantage) Quintile 2 Quintile 3 Quintile 4 Quintile 5 (least disadvantage)	*0.572 (0.386*–0.849)* *0.470 (0.246*–0.898)* 1.058 (0.692–1.617) 1.544 (0.994–2.398) *2.912 (1.641*–5.167)*	-
Australian Statistical Geography Standard (ASGS) Major city Inner regional Outer regional Remote Very remote	*1.806 (1.220*–2.674)* 0.944 (0.536–1.663) *0.582 (0.344*–0.984)* 0.856 (0.288–2.543) *0.096 (0.013*–0.730)*	-
Aetiology		
Assault Fall Sports RTA Work-related Animal-related Other	*0.014 (0.003*–0.056)* 34.738 (19.111–63.143)* *0.044 (0.006*–0.324)* *0.344 (0.157*–0.755)* 1.148 (0.190–6.933) *0.077 (0.010*–0.577)* #	*0.017 (0.004*–0.072)* *33.312 (17.413*–63.347)* *0.033 (0.004*–0.247)* *0.270 (0.117*–0.624)* 1.144 (0.180–7.263) *0.093 (0.012*–0.747)* #
Facial fractures Mandible Orbitozygomatic Orbit Zygomatic arch Nasal NOE Frontal sinus Dentoalveolar Panfacial	*0.424 (0.256*–0.702)* *2.493 (1.701*–3.652)* 1.073 (0.724–1.589) 0.506 (0.138–1.864) 0.657 (0.357–1.208) 0.426 (0.047–3.835) 0.422 (0.089–2.010) 0.484 (0.100–2.356) #	*0.397 (0.202*–0.781)* *2.070 (1.186*–3.613)* 1.106 (0.626–1.955) 0.741 (0.111–4.953) 1.039 (0.414–2.612) 0.467 (0.036–6.099) 0.864 (0.085–8.800) 0.376 (0.061–2.331)
Multiple fractures > 1 fracture	*0.537 (0.319*–0.905)*	0.875 (0.398–1.921)
Associated injuries	*2.625 (1.794*–3.840)*	*2.319 (1.267*–4.245)*
Cervical spine fracture	1.747 (0.603–5.060)	2.623 (0.434–15.846)
Recurrent fracture	0.612 (0.315–1.188)	0.727 (0.247–2.141)
Treatment		
Surgery	0.150 (0.095–0.238)*	0.443 (0.225–0.872)*
ORIF	0.225 (0.126–0.402)*	0.598 (0.236–1.516)
Complications	*0.393 (0.146*–*1.061)*	3.810 (0.783–18.533)
Re-operation	0.187 (0.023–1.486)	0.276 (0.012–6.434)

^*^Italicised values indicate statistical significance, *P* < 0.05; #, no numerical value

Almost all (92.6%) elderly facial fractures were from falls with an increased risk of 34.7 times compared to younger women. Younger women had significantly greater proportions of assaults, sports, RTA, and animal-related facial fractures (Table 2). The mandible and orbitozygomatic fractures represented half the facial fractures amongst younger women. Elderly women were 42.4% less likely to have a mandibular fracture, but 2.5 times more likely to have an orbitozygomatic fracture. Younger women had greater proportions for multiple fractures (19.7% vs 11.6%) with elderly women 53.7% less likely. Associated injuries were present in almost half the elderly with a 2.6 times risk compared to younger women. Younger women had higher incidences of surgical intervention (52.6% vs 14.3%) with elderly women 15.0% less likely compared to their counterparts. Multivariate analysis for surgery when accounted for ASGS, ISRAD, seasons, alcohol, facial fractures, and aetiology showed elderly women were 44.3% less likely to have surgery compared to younger women (Table 3). Post-operative complications showed a trend towards statistical significance with younger women having greater proportions compared to elderly (6.5% vs 2.6%, *P* = 0.057).

## Discussion

In South Australia, the three most vulnerable groups for facial trauma in females include young women related to assault, elderly women with falls, and indigenous women with disproportionately higher rates. An Irish study of 1190 female facial fractures reported peaks at 20–39 years and 70–89 years with the midface commonly afflicted [10], whereas Gerber’s British study reported peaks at 20–29 years and a third over 60 years caused mainly by accidents, assaults, and then falls [11]. The aetiology of facial fractures has changed over the decades and influenced heavily by sociocultural values and laws pertaining to the country. In the 1990s, RTA was reported as the most common cause of facial fractures in the USA for women, followed by assaults, with the mandible frequently afflicted [12]. Our previous work on mandibular fractures compared the aforementioned period to the present with stark differences: assaults (45% vs 35%), RTA (27% vs 6.8%), and falls (22% vs 43.7%) [13, 14]. Consistent with the literature [15–17], a bimodal age distribution for young and elderly women who present with facial trauma can assist surgeons in establishing a risk profile and management plan.

Younger women, notably 26–35 years, from lower socioeconomic backgrounds had higher rates of non-alcohol-related assaults and multiple fractures compared to elderly women. This is consistent with the national data on assaults reporting 56 cases per 100,000 population with the 30–34 age group most frequently affected [18]. There was an alarming proportion of non-alcohol-related assaults for women involving partners, family members, and/or friends. Retrospective analysis of the data did not consistently document the circumstances of the events as the victim often did not disclose exact details. In some cultures, it has been reported that assaulted females may provide inadequate documentation because of sociocultural reasons indirectly causing harm [19]. Arosarena’s study recognised patterns of assaults to where intimate partner violence was more likely to have zygomatic complex fractures, orbital blow-out fractures, and intracranial injuries, whereas women assaulted by unknown or unidentified assailants were more likely to have mandible fractures [20]. Our experience identified an overall higher proportion of midface than mandibular fractures (78 vs 36), but indigenous proportions were inversely affected (12 vs 9) suggesting the mechanism of injury and assault were different. One of the challenges in clinical history and assessment is the validity of sensitive information to distinguish between partners or unknown assailants. Nevertheless, if features are suggestive of domestic violence in vulnerable groups like younger women, indigenous women, or pregnant women, providing appropriate referral and supportive services should be simultaneously offered at the time of trauma assessment.

The profile of violence against women has been associated with degree of suburban living, education, marital status, and residency with higher rates for younger ethnic minority women [21–23]. We recognised two distinct groups of socioeconomic disadvantage where younger women from most disadvantaged areas had higher rates of injury from assault, but older women from least disadvantaged areas were 2.9 times more likely to have fall-related facial fractures (Table 2). Australian and New Zealand national data have identified twice the rate of injury for indigenous and even greater in remote areas compared to non-indigenous [24–27]. We similarly reported two trends where elderly women from major cities had 1.8 times the rate compared to younger women, but with increasing remoteness younger women, notably indigenous women, had higher risks of facial fractures largely from assaults. Oberdan’s mandibular study affirmed these findings with alarmingly high facial fractures in indigenous females from assaults with higher levels of recurrent trauma than non-indigenous [28]. In a younger indigenous population, alcohol and assault were more likely to result in facial fractures, whereas sport or fall-related facial fractures had higher rates in non-indigenous women. We have previously identified that indigenous people aged 26–35 years were 1.5 times more likely to have a facial fracture with greater rates in remote areas; they experienced higher rates of mandibular fractures with higher rates of operative intervention, post-operative complications, and extended LoS compared to non-indigenous women [29].

Falls are a common cause of injury in the elderly with highest incidences in developing countries compared to developing countries [21, 30, 31] A Japanese study on falls reported the mandible was commonly afflicted with an overall conservative approach compared to younger women (55.0% vs 86.4%) [32]. Our multivariate analysis showed that elderly women were 44.3% less likely to have surgery compared to younger women (14.3% vs 52.6%) with no difference in complications or re-operations. This is reflective of the difference in aetiology and facial fractures experienced amongst age groups where the midface was commonly afflicted from falls and assaults, respectively. Seasonality is an important contributor to the presentation of facial fractures often relating to warmer weather, outdoor activities, and frailty. A decade review of Chinese maxillofacial fractures identified an increase in trauma for females during summer with more frequent condylar fractures than men [33]. In contrast, we established elderly women were twice as likely to have a fall-related facial fracture during winter compared to younger women (Table 3). The impact of seasonality is seldom established in maxillofacial studies, as it is multifactorial with frailty, environmental hazards, and malnutrition superimposed on comorbidities and sociodemographic factors [34, 35]. With an older population commonly presenting with falls, associated injuries are more frequent and directly impact LoS (OR = 2.3). The rate and types of associated injuries differ depending on the mechanism such as orthopaedic secondary to RTA (23.2%) [36], neurological secondary to RTA (19.2%) [37], and soft tissue injuries from falls (67.6%) [38]. Our rate was 32.2% with the elderly 2.3 times more likely to have different types of associated injuries compared to younger females. The most common types of injuries for the elderly included cardiovascular, neurological, and upper limb injury, whereas younger women suffered soft tissue, neurological, and upper and lower limb injuries.

The operative rate was significantly higher in younger females attributed mainly by the mandible, whereas fall-related fractures in elderly females resulted in orbitozygomatic fractures managed non-operatively (OR = 0.443, Table 3). Sport is another popular growing subgroup that was increasingly common amongst younger females with disproportionate rates compared to men. Females aged 36–55 years have approximately five times the risk of a sport-related facial fracture principally from cycling, whereas males have decreasing risks of approximately 20% compared to 18–25 year olds [39]. Other studies with comparable demographic and fracture profiles have also reported similar operative rates [40]; however, we have further established significant risk profiling for elderly women with mandibular and orbitozygomatic fractures (OR = 0.424, OR = 2.493, Table 3). Operative intervention varies greatly depending on the fracture and age, which we generally favoured conservative measures for elderly women balancing comorbidities, function, and quality of life. With a higher operative rate amongst younger women, there was a trend to statistical significance to suggest higher post-operative complications reflective of the type of facial fracture (mandible vs orbitozygomatic).

This is the first statewide and national paper that presents a risk profile analysis for facial fractures in women providing the clinician with key socioeconomic parameters and clinical points. The three major vulnerable groups discussed highlight important aspects of assessment and intervention with facial trauma. Opportunities for government and community-based programs targeted for domestic violence and fall prevention is key to a growing elderly population. There are salient differences in aetiology and presentation compared to men that surgeons and trainees should incorporate in their assessment and management of maxillofacial fractures for young women and the elderly. Key limitations include retrospective and selection bias, however, future studies on differences in associated injuries would provide more insight into prevention and management. Ongoing surveillance for trends in women, particularly indigenous, would provide invaluable indicators of clinical outcomes and standard of service.

## Conclusion

Younger females are more likely to have mandibular fractures with operative intervention from assaults, whereas elderly females principally present with fall-related facial fractures with higher rates of associated injuries. Surgeons should employ a holistic risk profile assessment for women who present with facial trauma in their trauma service.

## Data Availability

The data cannot be shared due to ethics.

## Code availability

IBM SPSS V 26.0 was used for statistical analysis.
